# AKIR-1 regulates proteasome subcellular function in *Caenorhabditis elegans*

**DOI:** 10.1016/j.isci.2023.107886

**Published:** 2023-09-11

**Authors:** Johanna Pispa, Elisa Mikkonen, Leena Arpalahti, Congyu Jin, Carmen Martínez-Fernández, Julián Cerón, Carina I. Holmberg

**Affiliations:** 1 Department of Biochemistry and Developmental Biology, Medicum, Faculty of Medicine, University of Helsinki, 00290 Helsinki, Finland; 2 Department of Anatomy, Medicum, Faculty of Medicine, University of Helsinki, 00290 Helsinki, Finland; 3 Modeling Human Diseases in C. elegans Group, Genes, Diseases, and Therapies Program, Institut d'Investigació Biomèdica de Bellvitge - IDIBELL, L'Hospitalet de Llobregat, 08908 Barcelona, Spain

**Keywords:** Biochemistry, Genomics, Cell biology

## Abstract

Polyubiquitinated proteins are primarily degraded by the ubiquitin-proteasome system (UPS). Proteasomes are present both in the cytoplasm and nucleus. Here, we investigated mechanisms coordinating proteasome subcellular localization and activity in a multicellular organism. We identified the nuclear protein-encoding gene *akir-1* as a proteasome regulator in a genome-wide *Caenorhabditis elegans* RNAi screen. We demonstrate that depletion of *akir-1* causes nuclear accumulation of endogenous polyubiquitinated proteins in intestinal cells, concomitant with slower *in vivo* proteasomal degradation in this subcellular compartment. Remarkably, *akir-1* is essential for nuclear localization of proteasomes both in oocytes and intestinal cells but affects differentially the subcellular distribution of polyubiquitinated proteins. We further reveal that importin *ima-3* genetically interacts with *akir-1* and influences nuclear localization of a polyubiquitin-binding reporter. Our study shows that the conserved AKIR-1 is an important regulator of the subcellular function of proteasomes in a multicellular organism, suggesting a role for AKIR-1 in proteostasis maintenance.

## Highlights

• AKIR-1 is a conserved regulator of proteasome localization to the nucleus
• AKIR-1 loss induces subcellular redistribution of proteasomal degradation capacity
• AKIR-1 loss causes intestinal, but not oocytic, nuclear polyubiquitin accumulation
• AKIR-1 contributes to protein homeostasis *in vivo*

## Graphical abstract

**Figure:**
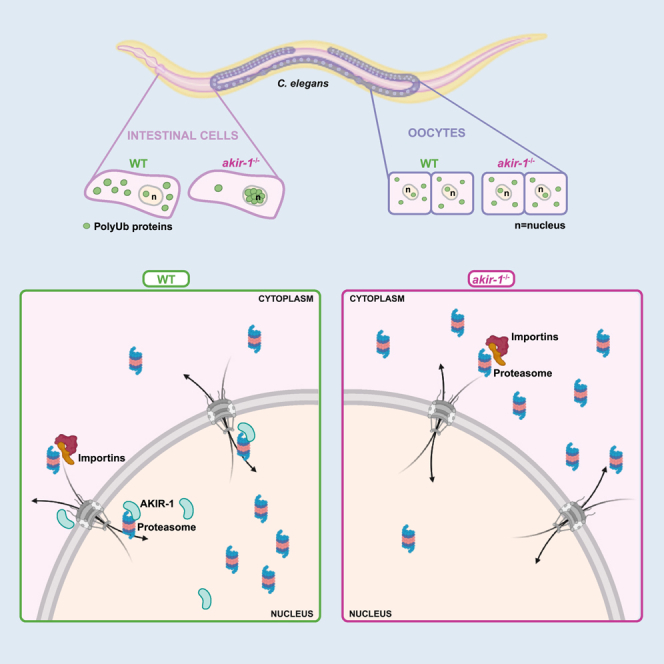

## Introduction

Protein degradation is one of the essential mechanisms that the cell uses to maintain its homeostasis and protein balance. The major machinery for degrading individual proteins is the ubiquitin-proteasome system (UPS), by which a cascade of enzymes for ubiquitin activation (E1), conjugation (E2), and ligation (E3) marks selected proteins with polyubiquitin chains as a signal for degradation.^1^ Hydrolysis of the tagged proteins into small peptide fragments is performed by the proteasome, a large 2.5-megadalton (MDa) protein complex composed of two subcomplexes: a catalytic core particle (CP or 20S) and a regulatory particle (RP or 19S). The catalytic activities, i.e., trypsin-, chymotrypsin- and caspase-like activity of the barrel-shaped 20S particle, are contained within the two seven-subunit β-rings, which are stacked between two seven-subunit α-rings. The CP can be capped at either one (26S) or both ends by the multisubunit regulatory particle, which functions in recognition, binding, deubiquitination, unfolding, and translocation of the substrates into the lumen of the CP.^2^ The proteasomal degradation capabilities are broadened with the ability of the free CP to degrade some proteins independently of ubiquitination^3^ and the existence of proteasome variants with alternative subunits of the CP or the regulatory particle, or activating complexes.^4^

During the last decades, studies based on biochemical and immunological methods and live-cell imaging have reported proteasomes to be localized both in the cytoplasm and in the nucleus in various cell types.^5^^,^^6^ Despite vast accumulating evidence on variation in nuclear and cytoplasmic distribution of proteasomes in different cell lines and physiological conditions, the regulatory mechanisms governing nuclear localization and function of the proteasomes are only beginning to emerge.^6^ Generally, controlled UPS-mediated nuclear degradation is important for the regulation of many nuclear proteins, such as several transcription factors, histones, or epigenetic modifying enzymes.^7^^,^^8^^,^^9^ However, there are also reports both in yeast and human cells showing that nuclei may lack proteolytic activity, suggesting that proteasomes localize mainly on the cytoplasmic side of the nuclear membrane.^10^^,^^11^ In addition, in some circumstances ubiquitinated proteins seem to be exported from the nucleus to the cytoplasm for degradation.^12^

Both in mitotic or post-mitotic cells, proteasomes or their subcomplexes are transported to the nucleus via the nuclear pores.^13^^,^^14^^,^^15^^,^^16^ For nuclear transport, receptors of the karyopherin beta family are generally required.^17^ Some of these receptors have been shown to be involved in the import of proteasomes: the importin α family member SRP1 in yeast,^18^ importin 5 in human melanoma and adenocarcinoma cells,^15^ and importin 9 both in human colon carcinoma cells^19^ and in *Drosophila melanogaster (D. melanogaster)* during spermatogenesis.^20^ In addition to importins, some adapter proteins have been found to mediate proteasome nuclear transport. In *Saccharomyces cerevisiae (S. cerevisiae) and Schizosaccharomyces pombe (S. pombe),* Sts1 and Cut8, respectively, bind proteasomes and are required for their nuclear import.^21^^,^^22^^,^^23^^,^^24^^,^^25^ Additionally, the alternative regulatory protein Blm10 has been identified as an adapter in nuclear import of CPs in yeast.^26^ In vertebrates, de Almeida and colleagues recently discovered human AKIRIN2 as a proteasome binding protein essential for the nuclear localization of proteasomes in cancer cell lines,^19^ but whether this is true for a broad range of cell types as found in an entire organism remains to be elucidated.

Akirins are a highly conserved, metazoan-specific family of small nuclear localization signal (NLS)-containing nuclear proteins. The number of Akirin paralogues within species varies from one to eight, with *Caenorhabditis elegans* (*C. elegans)* having one copy, and vertebrates having two, AKIRIN1 and AKIRIN2.^27^^,^^28^ Akirins have been best known for their role in innate immunity, where they are required for expression of downstream effector genes in several organisms.^27^ The regulation of transcription is mediated by their interaction with chromatin remodeling complexes.^28^ In addition, Akirins are involved in developmental processes.^28^ *C. elegans* AKIR-1 has been shown to interact with chromatin remodeling complexes (Nucleosome Remodeling and histone Deacetylases NuRD I and II, and MEC, a *Drosophila* dMec homologue) to coordinate innate immunity responses, but also to regulate muscle development and function, and meiosis.^29^^,^^30^^,^^31^ Further, *C. elegans* AKIR-1 has also been shown to interact with nuclear importins, such as IMA-3 and IMA-2.^30^^,^^32^ The ability of human AKIRIN2 to regulate proteasome subcellular localization is a novel function for Akirins and appears not to involve transcriptional regulation.^19^

We have previously demonstrated that proteasomes are localized both in the cytoplasm and in the nucleus in multiple tissues in *C. elegans*.^33^ Here, we show that the *C. elegans* gene *akir-1*, a homolog of human AKIRIN2, is required for the nuclear localization of proteasomes in *C. elegans* and that depletion of *akir-1* causes nuclear accumulation of polyubiquitinated proteins in a tissue-specific manner. In addition, downregulation of the *C. elegans* importins *ima-3* and *imb-1* mimics the *akir-1* RNAi-induced polyubiquitin phenotype in intestinal cells. Our results show that the function of Akirin proteins in proteasome nuclear transport is conserved between vertebrate and invertebrate cells and further extend information on the complex roles of AKIR-1 in a multicellular organism.

## Results

### Loss of akir-1 results in nuclear accumulation of polyubiquitinated proteins in C. elegans intestinal cells

**Figure 1:**
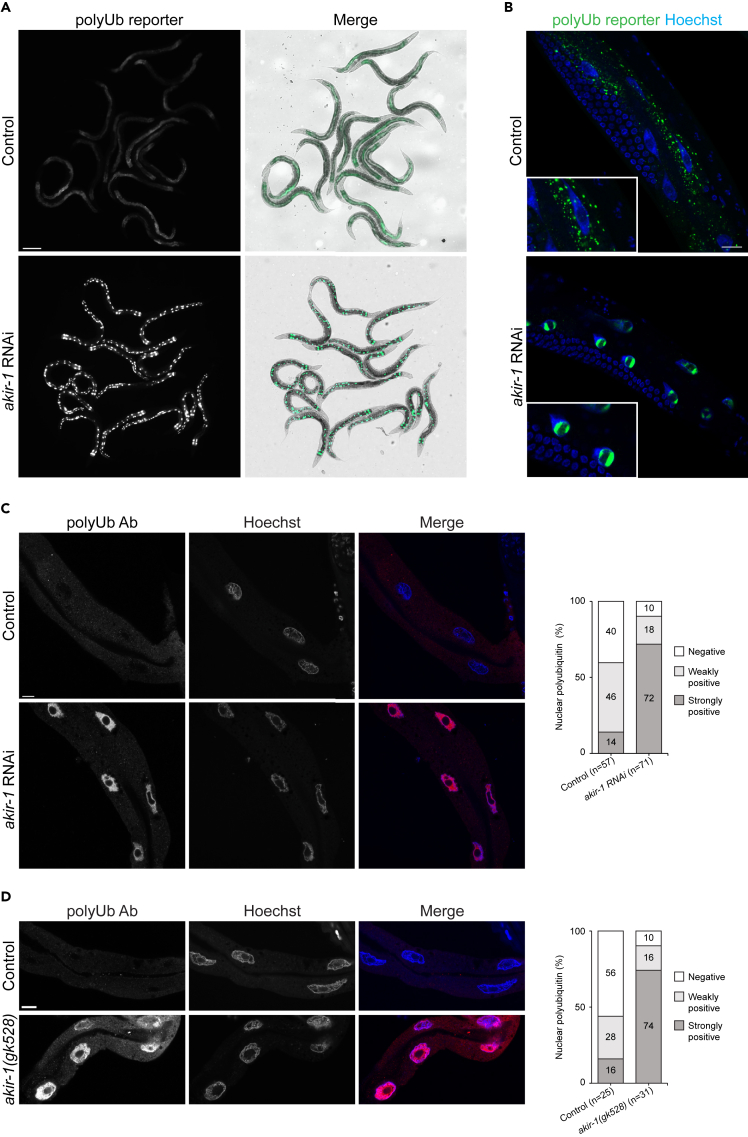
Loss of akir-1 results in nuclear accumulation of polyubiquitinated proteins in C. elegans intestinal cells (A) Representative fluorescence micrographs of control and *akir-1* RNAi-treated N2 animals expressing the polyubiquitin (polyUb) reporter (*vha-6p::UIM2-ZsProSensor*) in the intestinal cells (left panels). Merge represents overlay of fluorescence and bright-field images (right panels). Scale bar, 200 μm. (B) Representative confocal micrographs of control and *akir-1* RNAi-treated polyubiquitin reporter animals with Hoechst-visualized nuclei. Insets show enlargements. Scale bar, 20 μm. (C and D) Representative confocal micrographs of polyubiquitin immunostaining (polyUb Ab) in dissected intestines of control and *akir-1* RNAi-treated wild-type (N2) animals (C), and in control (N2) animals and *akir-1(gk528)* mutants (D). Nuclei are visualized with Hoechst. Scale bars, 10 μm. The graphs (on right) show visually quantified nuclear polyubiquitin accumulation in the intestine in control and *akir-1* RNAi-treated wild-type animals (C), and in control (N2) and *akir-1(gk528)* animals (D). n = total number of animals. Proportions of animals with strongly positive, weakly positive, or negative (i.e., less or equal to cytoplasmic immunostaining) nuclear immunostaining are indicated in percentages. See also Figure S1.

We have previously created a transgenic *C. elegans* strain expressing a polyubiquitin-binding fluorescent reporter in intestinal cells to facilitate *in vivo* analysis of endogenous polyubiquitinated proteins.^34^ The reporter is composed of the short-lived fluorescent ZsProSensor protein, which is degraded by the proteasome in an ubiquitin-independent manner, fused to ubiquitin-interacting motifs (UIMs) derived from the RPN-10 ubiquitin receptor.^34^ This reporter binds endogenous polyubiquitinated proteins via its UIMs resulting in a fluorescent phenotype of the reporter strain.^34^ The polyubiquitin-binding reporter’s stability, as reflected by its fluorescence, is affected by the balance between proteasomal degradation and binding to polyubiquitinated proteins. Increased fluorescence reflects stabilization of the reporter upon accumulation of endogenous polyubiquitinated proteins, and we have previously shown that the reporter responds to changes in proteasome activity and physiological stimuli.^34^^,^^35^^,^^36^ We performed a genome-wide RNAi screen using these intestinal polyubiquitin-binding reporter animals, which were subjected to standard feeding RNAi from L1 larval stage and scored at day 1 of adulthood. We identified *akir-1* (*E01A2.6*) as one hit gene, out of more than 30 potential new regulators of the proteasome (screen data not included here), displaying a distinct phenotype. We discovered that downregulation of *akir-1* both increased the reporter fluorescence (mean fold induction 4.5 ± SD 2.5, n = 3 independent experiments) and strikingly changed its uniform and diffuse cellular distribution into a more converged pattern (Figure 1A). Hoechst staining of DNA confirmed that the new fluorescence pattern resulted from the polyubiquitin-binding reporter concentrating mainly into the intestinal nuclei, excluding the nucleoli (Figure 1B), of *C. elegans*.

Our results with the polyubiquitin-binding reporter strain suggest that *akir-1* downregulation causes accumulation of endogenous polyubiquitinated proteins in intestinal nuclei. We next used an anti-polyubiquitin antibody for immunostaining of dissected intestines from wild-type N2 animals treated with control or *akir-1* RNAi. We used dissected tissues to improve antibody tissue penetrance during immunostaining. Although both control and *akir-1* RNAi-treated animals showed positive nuclear staining of polyubiquitinated proteins in the intestine, the portion of animals with positive nuclear staining clearly increased from 60% to 90% upon *akir-1* RNAi (Figure 1C). Importantly, when the accumulating polyubiquitin immunostaining in the intestine nuclei was visually estimated as none (i.e., less or equal to cytoplasmic immunostaining), weak, or strong, the number of animals with strong immunostaining increased from 14% in control to 72% upon *akir-1* RNAi treatment (Figure 1C). As qPCR analysis showed that *akir-1* mRNA was downregulated efficiently, but not completely, after *akir-1* RNAi treatment (Figure S1A); we examined polyubiquitinated proteins also in the *akir-1(gk528)* deletion mutant, which is predicted to be a null allele.^29^ In accordance with the *akir-1* RNAi results, *akir-1* mutants displayed a clear increase in nuclear polyubiquitin immunostaining compared to wild-type animals (90% *akir-1(gk528)*, 44% control), and, in particular, the strong nuclear immunostaining increased from 16% in wild-type animals to 74% in *akir-1* mutants (Figure 1D). To investigate the total levels of endogenous polyubiquitinated proteins, we performed western blot analysis of whole animal lysates with an anti-polyubiquitin antibody. Variability among independent experiments was observed, but no systematic difference in the amount of polyubiquitinated proteins was detected in lysates of *akir-1* mutants compared to control lysates (Figure S1B). Taken together, our results show that depletion of *akir-1* leads to accumulation of polyubiquitinated proteins in the nucleus of intestinal cells in *C. elegans*.

### Knockdown of akir-1 alters proteasome activity

**Figure 2:**
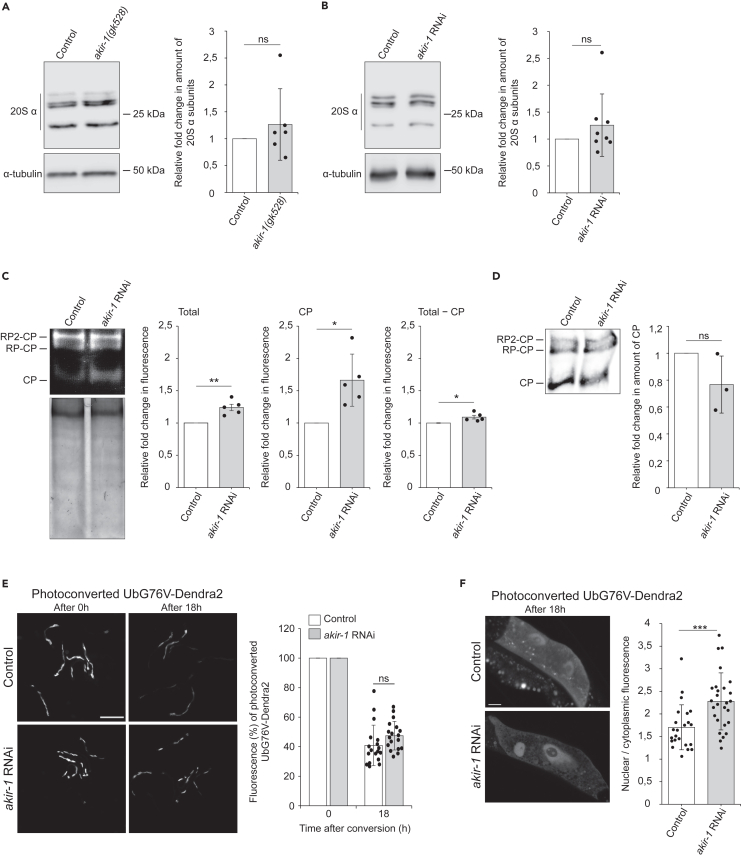
Knockdown of akir-1 alters proteasome activity (A and B) Western blot analysis with antibody against proteasomal 20S alpha subunits using lysates of *akir-1(gk528)* mutants and control (N2) animals (A), and of control and *akir-1* RNAi-treated wild-type animals (B). Anti-alpha-tubulin antibody was used as a normalization control. The quantification graphs show the average fold change in *akir-1(gk528)* mutants compared to control (N2) animals (set to 1; n = 6 independent experiments) (A), and in *akir-1* RNAi-treated wild-type animals compared to control RNAi-treated animals (set to 1; n = 8 independent experiments) (B). (C) In-gel proteasome activity assay with whole animal lysates of wild-type animals exposed to control or *akir-1* RNAi treatment (upper gel). Coomassie staining of the same gel (lower gel). The quantifications show the average fold change in chymotrypsin activity in *akir-1* RNAi-treated wild-type animals compared to control RNAi-treated animals (set to 1; n = 5 independent experiments). Proteasome activity is indicated as total (Total; CP + RP-CP + RP2-CP), as core particle (CP), and as CP activity subtracted from total activity (Total − CP). RP, Regulatory particle. (D) Immunoblot analysis of whole animal lysates separated under non-denaturing condition using the antibody against proteasomal 20S alpha subunits. Ponceau S staining was used for total protein normalization. The quantification graph shows the average fold change of core particles (CP) in *akir-1* RNAi-treated wild-type animals compared to control RNAi-treated animals (set to 1; n = 3 independent experiments). (E) Representative fluorescence micrographs of control and *akir-1* RNAi-treated transgenic *C. elegans* expressing photoconvertible UbG76V-Dendra2 reporter (*vha-6p::UbG76V::Dendra2*) in intestinal cells. The 0 h (left panels) and 18 h (right panels) indicate time after photoconversion. Scale bar, 500 μm. The graph shows the mean percentages of fluorescence intensity of the photoconverted UbG76V-Dendra2 18 h after the photoconversion relative to the fluorescence at the point of photoconversion (0 h, set as 100%); n = 6 independent experiments with triplicate images of 6–7 animals per image (total number of animals is 108 per treatment). (F) Representative confocal fluorescence micrographs of intestinal cells with photoconverted UbG76V-Dendra2 (18 h after conversion) in transgenic *C. elegans* treated with control or *akir-1* RNAi. Scale bar, 10 μm. The graph shows the ratio between nuclear and cytoplasmic mean fluorescence per cell. n = 2 independent experiments (total number of nuclei is 23 in control RNAi and 27 in *akir-1* RNAi treatment). Welch’s t-test (two-tailed distribution and unequal variance) was used for statistical analyses. Error bars, SD; ns, not significant; ∗p < 0,05; ∗∗p < 0,01; ∗∗∗p < 0.001. See also Figure S2.

Accumulation of polyubiquitinated proteins is commonly caused by altered proteasome levels or activity. We first analyzed the total amount of endogenous proteasomes in whole animal lysates by western blot analysis using an antibody against 20S alpha subunits. No clear change in 20S levels was detected in lysates of either *akir-1* mutants (Figure 2A) or *akir-1* RNAi-treated animals (Figure 2B) compared to control animals. Next, we performed an in-gel proteasome activity assay on whole animal lysates and detected a slight increase in total proteasome activity upon *akir-1* RNAi (Figure 2C, left graph). Interestingly, the 20S CP appears to be the main contributor of this increased activity (Figure 2C). To test whether the increased CP activity after *akir-1* RNAi is caused by changes in the amount of CP complexes, we analyzed the CP levels by immunoblotting native gels with the antibody against 20S alpha subunits. No clear change in the levels of the 20S CPs was detected in *akir-1* RNAi-treated animals compared to control RNAi-treated animals (Figure 2D). To specifically measure proteasome activity in intestinal cells of *C. elegans*, we employed our previously established UPS reporter animals expressing the photoconvertible proteasomal substrate UbG76V-Dendra2 in the intestine.^37^^,^^38^ In these animals, a decrease in the amount of photoconverted UbG76V-Dendra2 reporter reflects *in vivo* proteasome activity. We first measured the fluorescence of the photoconverted UbG76V-Dendra2 in the whole intestine and detected a similar rate of degradation in control and *akir-1* RNAi-treated animals (Figure 2E, extended in Figure S2). As *akir-1* depletion leads to nuclear accumulation of polyubiquitinated proteins, we next monitored photoconverted UbG76V-Dendra2 fluorescence intensity at single-cell level, specifically in the nucleus and cytoplasm, in living animals. The fluorescence of UbG76V-Dendra2 at 18 h after photoconversion was more intense in the intestinal nuclei than in the cytosol upon *akir-1* RNAi (Figure 2F), demonstrating that *akir-1* knockdown results in slower proteasomal degradation in intestinal nuclei *in vivo.* Taken together, our results suggest that AKIR-1 influences subcellular proteasome activity.

### Intestinal nuclei display reduced proteasome levels upon akir-1 depletion

**Figure 3:**
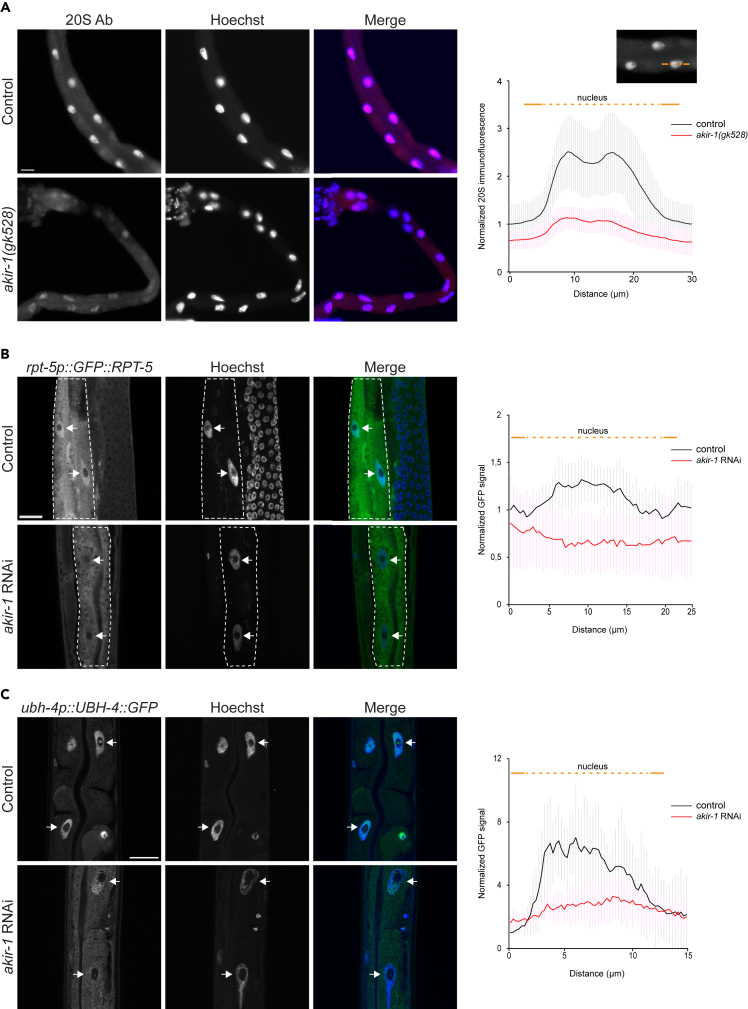
Intestinal nuclei display reduced proteasome levels upon akir-1 depletion (A) Representative micrographs of proteasome immunostaining (20S Ab) in dissected intestines of control (N2) animals and *akir-1(gk528)* mutants. The graph shows the normalized mean ± SD intensity profiles of 20S immunofluorescence measured along the line intersecting the cytoplasm and the nucleus as shown in the image above the graph. Orange line represents the profiling line, dashed line represents the nucleus. During the profiling, Hoechst signal was used to determine the nuclear 20S immunofluorescence. n = total number of nuclei is 26 in control (N2) and 44 in *akir-1(gk528)* animals. (B) Representative confocal micrographs showing GFP fluorescence ratio between nuclei and cytoplasm of control and *akir-1* RNAi-treated *rpt-5p::GFP::RPT-5* animals. Intestinal cells are outlined with white dashed lines and white arrows point to intestinal cell nuclei (C) Representative confocal micrographs of *ubh-4p::UBH-4::GFP* animals. Nuclei are visualized with Hoechst. The graphs show the normalized mean ± SD intensity profiles of fluorescence measured along the line intersecting the cytoplasm and the nucleus. n = total number of nuclei is 13 in control RNAi and 17 in *akir-1* RNAi treatment (B) and 8 nuclei in control RNAi and 9 nuclei in *akir-1* RNAi treatment (C). Scale bars, 20 μm. Error bars, SD. See also Figure S3.

As the accumulation of polyubiquitinated proteins and the altered *in vivo* proteasome activity in the intestine of *akir-1* mutant and RNAi-treated animals occur specifically in the nucleus, we investigated subcellular distribution of the proteasome by immunostaining dissected intestines with the anti-20S antibody. We have previously shown using this antibody that the proteasome is concentrated in nuclei in the intestine of wild-type *C. elegans*.^33^ Consistently, dissected intestines of wild-type animals displayed high immunofluorescence in the nuclei (Figures 3A and S3). Compared to the controls, both *akir-1* mutants and *akir-1* RNAi-treated animals showed a consistent reduction in 20S immunofluorescence intensity in intestinal nuclei, when we measured immunofluorescence profiles along a line intersecting the cytoplasm and the nucleus (Figures 3A and S3). As a complementary approach, we investigated the proteasome subcellular localization using transgenic *C. elegans* expressing extrachromosomal arrays of GFP-tagged RPT-5, a subunit of the 19S RP particle of the proteasome, under its endogenous *rpt-5* promoter. The transgenic animals showed a mosaic fluorescence pattern of the ubiquitously expressed GFP::RPT-5. GFP::RPT-5 animals displayed a stronger nuclear fluorescence compared to the cytoplasmic fluorescence in intestinal cells, and upon *akir-1* RNAi a clear reduction in nuclear fluorescence was also observed (Figure 3B), indicating nuclear decrease of 26S proteasome in the intestine. In addition, we investigated the localization of the proteasome-associated deubiquitinase (DUB) UBH-4. UBH-4, and its human homolog UCHL5, have previously been shown to interact with the 19S subunit RPN-13^34^^,^^39^^,^^40^^,^^41^ and to be broadly expressed in *C. elegans* tissues, including the intestine.^34^^,^^42^ Here, we used a CRISPR-engineered UBH-4:GFP strain^42^^,^^43^ and observed that these animals displayed strong nuclear fluorescence in intestinal nuclei similar to the pattern detected with the anti-20S antibody (Figures 3C and 3A). In accordance with the 20S immunofluorescence and GFP::RPT-5 results, UBH-4:GFP fluorescence decreased in the intestinal nuclei of animals exposed to *akir-1* RNAi when compared to control animals (Figure 3). Together, these results demonstrate that proteasome levels decrease in intestinal nuclei upon the loss of AKIR-1.

### Depletion of akir-1 affects nuclear polyubiquitinated proteins differently in oocytes and body-wall muscle cells compared to intestinal cells

**Figure 4:**
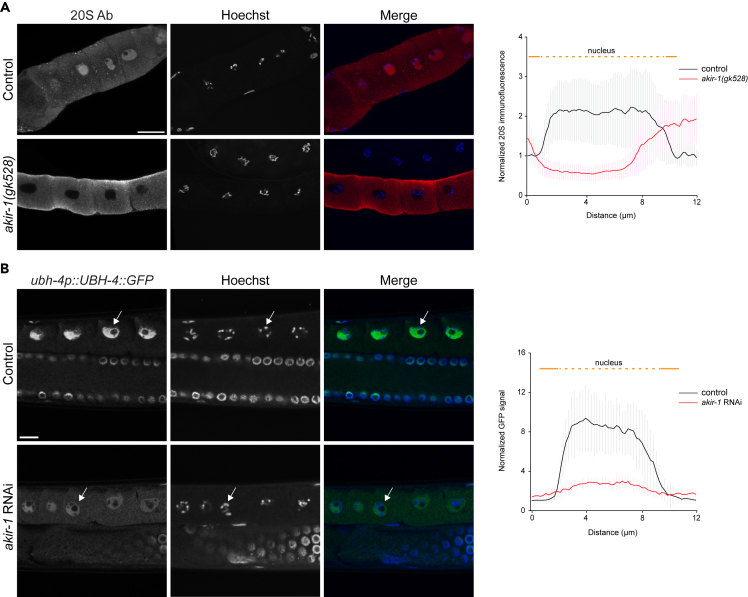
Nuclear proteasome expression decreases in oocytes upon akir-1 depletion (A) Representative confocal micrographs of proteasome immunostaining (20S Ab) in dissected oocytes of control (N2) animals and *akir-1(gk528)* mutants. The graphs show the normalized mean ± SD intensity profiles of 20S immunofluorescence measured along the line intersecting the cytoplasm and the nucleus. n = total number of nuclei is 10 in control (N2) and 11 in *akir-1(gk528)* animals. (B) Representative confocal micrographs showing GFP fluorescence in oocytes of control and *akir-1* RNAi-treated *ubh-4p::UBH-4::GFP* animals. The graph shows the normalized mean ± SD intensity profiles of fluorescence measured along the line intersecting the cytoplasm and the nucleus. n = total number of nuclei is 14 in control RNAi and 10 in *akir-1* RNAi treatment. White arrows point to oocyte nuclei. Nuclei are visualized with Hoechst. Scale bars, 10 μm. Error bars, SD. See also Figure S4.

**Figure 5:**
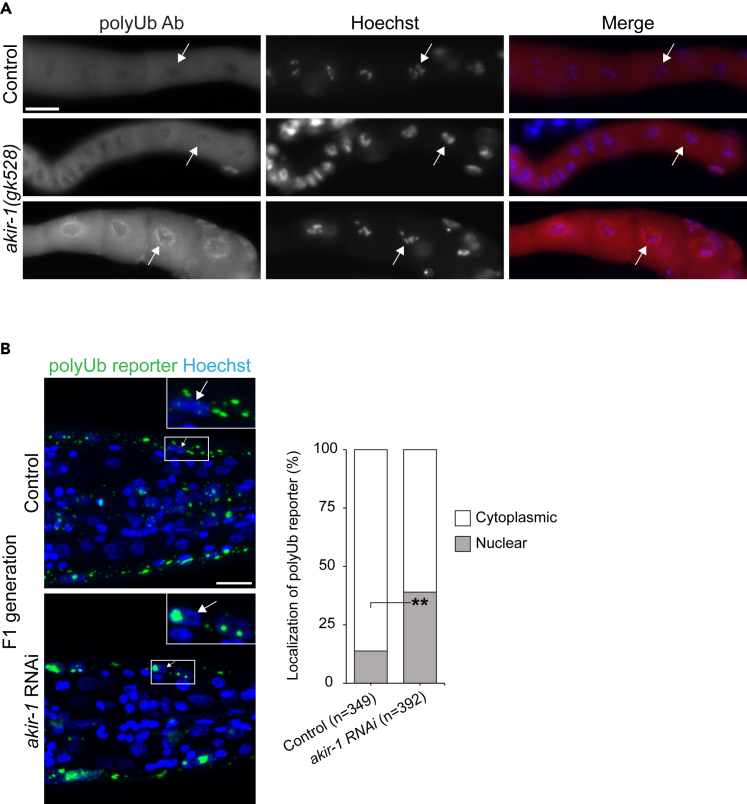
The effects of akir-1 depletion on nuclear accumulation of polyubiquitinated proteins in oocytes and body-wall muscle cells (A) Representative micrographs of polyubiquitin immunostaining (polyUb Ab) in dissected oocytes of control (N2) animals and *akir-1(gk528)* mutants. Nuclei are visualized with Hoechst. Scale bar, 20 μm. White arrows point to representative nuclei. (B) Representative fluorescence micrographs of F1 generation of control and *akir-1* RNAi-treated *rrf-3(pk1426)* animals expressing the polyubiquitin (polyUb) reporter (*unc-54p::UIM2-ZsProSensor*) in the body-wall muscle cells. Nuclei are visualized with Hoechst. Insets show enlargements of the indicated areas. White arrows point to representative nuclei. Scale bar, 10 μm. The graph (on right) shows quantified subcellular localization of polyUb reporter fluorescence in the body-wall muscle cells. Welch’s t-test (two-tailed distribution and unequal variance) was used for statistical analysis. n = total number of nuclei; ∗∗p < 0,01. See also Figure S5.

While performing 20S immunostaining with dissected *akir-1* mutants, we noticed that loss of *akir-1* altered proteasome subcellular distribution not only in the intestinal cells but also in the oocytes. Oocytes of wild-type animals displayed cytoplasmic and strong nuclear 20S immunofluorescence (Figures 4A and S4). Compared to the wild-type animals, nuclear proteasome localization is clearly reduced in oocytes of *akir-1* mutants (Figure 4A). A similar decrease in intensity of nuclear 20S immunostaining was also detected in oocytes of animals exposed to *akir-1* RNAi (Figure S4). Further, analysis of UBH-4::GFP animals treated with *akir-1* RNAi confirmed reduced levels of nuclear proteasomes in oocytes (Figure 4B). Taken together, these results show that the depletion or downregulation of *akir-1* causes a decrease in nuclear proteasomes in oocytes, similarly to our observation in intestinal cells. However, despite the clear reduction in nuclear proteasomes, we did not observe accumulation of polyubiquitinated proteins in the oocyte nuclei of *akir-1* mutants (Figure 5A). Most oocytes showed a relatively uniform cytoplasmic polyubiquitin immunofluorescence pattern, with weaker signal in the nuclei of both control animals and *akir-1* mutants (Figure 5A). Occasionally, some polyubiquitin-positive staining was detected at the rim of the nuclear membrane in *akir-1*-depleted animals (Figure 5A, lower panel). The polyubiquitin staining pattern in oocytes of *akir-1* RNAi-treated animals resembled the results of *akir-1* mutants (Figures 5A and S5A). Importantly, our results revealed that polyubiquitinated proteins do not accumulate to a similar degree in oocyte nuclei, as in intestinal nuclei, upon loss of *akir-1*.

AKIR-1 is required for *C. elegans* muscle development, integrity, and function.^31^ Therefore, we tested the impact of the loss of *akir-1* on polyubiquitinated proteins in muscles using our previously established transgenic strain expressing the polyubiquitin-binding reporter in the body-wall muscle cells.^35^^,^^36^ Quantification of the mean fluorescent intensity of the polyubiquitin-binding reporter in live animals showed no difference between control and *akir-1* RNAi treatments either in wild-type (N2) background (mean fold change 1.15 ± SD 0.19, n = 3 independent experiments) or in the RNAi-sensitive *rrf-3* background (mean fold change 0.94 ± SD 0.09, n = 2 independent experiments), and no difference in the fluorescence pattern itself was observed (Figure S5B). We also assessed proteasome activity using the photoconvertible UbG76V-Dendra2 reporter expressed in body-wall muscle cells of *C. elegans*.^37^ No difference in the degradation of photoconverted UbG76V-Dendra2 was detected in *akir-1* RNAi-treated animals compared to control RNAi-treated animals (Figure S5C).

As the RNAi experiments were performed by placing stage 1 larvae (L1) on RNAi plates and assessing the phenotype at day 1 of adulthood, we investigated whether remaining maternal AKIR-1 contribution might influence the lack of polyubiquitin accumulation in body-wall muscle cells. To this end, polyubiquitin-binding reporter animals (in *rrf-3* background) were continuously exposed to *akir-1* RNAi, and their F1 offspring treated in similar manner were monitored at day 1 of adulthood. We assigned reporter fluorescence signals either as nuclear or non-nuclear, based on colocalization with Hoechst nuclear staining. A slight increase in nuclear localization of the reporter was detected in *akir-1* RNAi-treated F1 animals (control 14%, *akir-1* RNAi 39% nuclear signal) (Figure 5B). Taken together, our results suggest that body-wall muscle cells respond differently to *akir-1* depletion in terms of nuclear accumulation of polyubiquitinated proteins than oocytes or intestinal cells.

### Perturbed nuclear transport mimics the akir-1 RNAi-induced polyubiquitin phenotype

**Figure 6:**
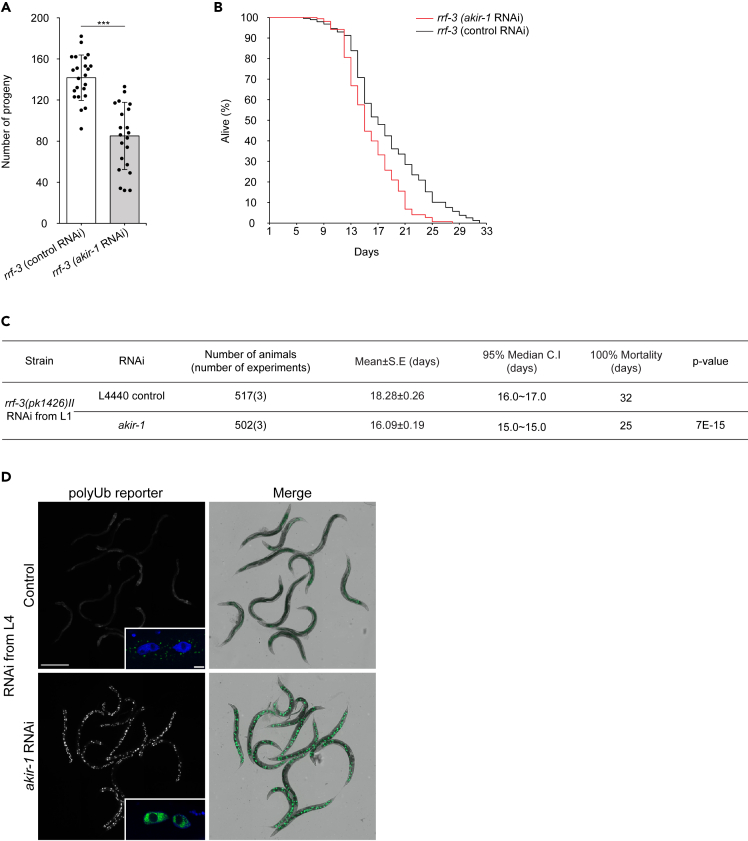
Downregulation of akir-1 reduces progeny number and lifespan, and promotes nuclear accumulation of polyubiquitin-binding reporter in intestinal cells even during adulthood (A) The graph shows the mean number of progeny of *rrf-3(pk1426)* animals exposed to control or *akir-1* RNAi treatment. n = 3 independent experiments (total number of P0 animals is 23 for control RNAi and 22 for *akir-1* RNAi). Welch’s t-test (two-tailed distribution and unequal variance) was used for statistical analysis. Error bars, SD; ∗∗∗p < 0.001. (B) A representative Kaplan-Meier survival curve from one experiment with *rrf-3(pk1426)* animals exposed to control or *akir-1* RNAi treatment started at the L1 larval stage. (C) The table shows the statistics of three independent lifespan experiments of *rrf-3(pk1426)* animals treated with control or *akir-1* RNAi started at the L1 larval stage, and significance of *akir-1* RNAi treatment compared to control RNAi treatment determined with a Mantel-Cox (log rank) test. Mean, restricted mean survival; C.I, confidence interval. (D) Representative fluorescence micrographs of 3-day adult animals expressing the polyubiquitin (polyUb) reporter in intestinal cells (*vha-6p::UIM2-ZsProSensor*) and treated with control or *akir-1* RNAi (left panels) started at the L4 larval stage. Merge represents overlay of fluorescence and bright-field images (right panels). Scale bar, 500 μm. Insets on left panels show representative confocal micrographs of polyubiquitin reporter animals with Hoechst-visualized nuclei. Fluorescence signal in the insets micrographs have been modified differently to show the most representative fluorescence pattern in both treatments. Scale bar, 10 μm.

Akirin proteins have been implicated in several physiological processes.^28^ In *C. elegans,* AKIR-1 is required for proper meiosis and development but is not essential for survival of the animal.^29^^,^^30^^,^^31^ To investigate phenotypes induced by *akir-1* downregulation under our experimental conditions, we compared the progeny number and lifespan of *rrf-3* animals treated with *akir-1* or control RNAi. We observed a 40% reduction in the number of progeny (Figure 6A) and a 2-day decrease in mean lifespan (Figures 6B and 6C) upon *akir-1* downregulation, which are in agreement with previous reports on the *akir-1* mutant and an epidermis-specific RNAi strain.^29^^,^^30^ To exclude that our detected effects on polyubiquitinated proteins and the proteasome are not linked to developmental defects, or to the completion of intestinal cell divisions in L1 larval stage,^44^ we also performed *akir-1* RNAi treatment starting from the L4 larval stage. These intestinal polyubiquitin-binding reporter animals, examined at day 3 of adulthood, displayed the same distinctive converged fluorescent pattern (Figure 6D), as detected when RNAi treatment was started at L1 larval stage (Figure 1A).

Previously, Akirins and AKIR-1 have been shown to act in the regulation of transcription via chromatin remodeling complexes.^28^ Therefore, we examined whether transcriptional regulation is required for the ability of AKIR-1 to affect proteasome function. In *C. elegans* AKIR-1 interacts specifically with the NuRD (NuRD I, II and MEC) chromatin remodeling complexes,^30^^,^^31^ which have been reported to display some subunit overlap (Figure S6A).^30^^,^^45^ These chromatin remodeling complex genes were not recognized as hits in our genome-wide RNAi screen, which could have been due to differences in experimental setup and fluorescence detection between the genome-wide scale and select gene RNAi, or due to the developmental criteria followed in our genome-wide RNAi screen. Thus, we next performed downregulation by select gene RNAi for each member of the NuRD complexes and MEC complex on the intestinal polyubiquitin-binding reporter animals but detected no phenotypes mimicking the *akir-1* RNAi-induced effect (Figure S6B). To directly affect transcription, we also performed RNAi against *ama-1*, a subunit of the RNA polymerase II, and while we observed a growth phenotype, the localization of polyubiquitin-binding reporter was not affected (Figure S6C).

**Figure 7:**
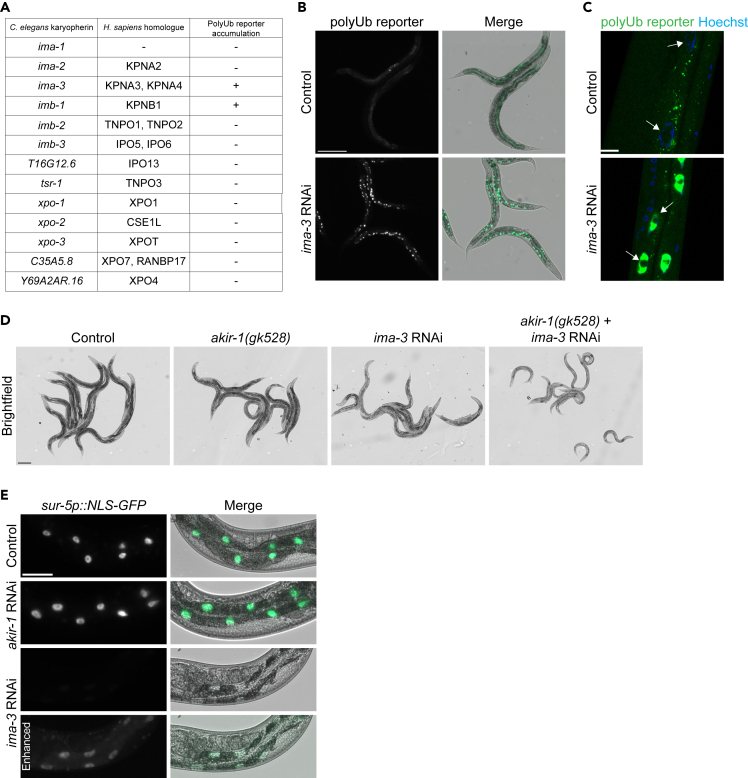
Distinctly perturbed nuclear transport mimics the akir-1 RNAi-induced polyubiquitin phenotype, but akir-1 is not required for general nuclear import (A) The table shows *C. elegans* karyopherins, their human orthologues, and RNAi-induced nuclear accumulation of the intestinal polyubiquitin (polyUb) reporter. (B) Representative fluorescence micrographs of control and *ima-3* RNAi-treated N2 animals expressing the polyubiquitin (polyUb) reporter (*vha-6p::UIM2-ZsProSensor*) in the intestinal cells (left panels). Merge represents overlay of fluorescence and bright-field images (right panels). Scale bar, 200 μm. (C) Representative confocal micrographs of control and *ima-3* RNAi-treated polyubiquitin reporter animals with Hoechst-visualized nuclei. White arrows point to representative intestinal cell nuclei. Scale bar, 10 μm. (D) Representative bright-field images of control (N2) and *akir-1(gk528)* mutant animals treated with control or *ima-3* RNAi, respectively. Scale bar, 200 μm. (E) Representative fluorescence micrographs of control, *akir-1*, and *ima-3* RNAi-treated wild-type animals expressing ubiquitiously nuclear-localized GFP (*sur-5p::NLS-GFP*) (left panels). Merge represents overlay of fluorescence and bright-field images (right panels). Lowest panels show enhanced fluorescence signal. Scale bar, 50 μm. See also Figure S7.

As the human homolog of AKIR-1, AKIRIN2, has recently been shown to mediate the nuclear import of proteasomes in conjunction with the cargo receptor, importin 9,^19^ and as interactions between AKIR-1 and importins have been reported,^30^^,^^32^ we tested whether *C. elegans* importins are similarly involved in regulating nuclear proteasomes. In *C. elegans*, 13 members of the karyopherin family have been identified (Figure 7A).^46^ We performed RNAi against all these karyopherin family members using our intestinal polyubiquitin-binding reporter strain (Figures 7B and S7). Of these, downregulation of importin α3, *ima-3*, showed clearly a similar polyubiquitin phenotype as *akir-1* RNAi (Figure 7B), although the intestinal fluorescent puncta formed with weaker intensity. DNA staining with Hoechst confirmed that the polyubiquitin-binding reporter concentrated to the intestinal nuclei upon *ima-3* RNAi treatment (Figure 7C). In addition, downregulation of importin β1, *imb-1*, partially mimicked the *akir-1* RNAi-induced fluorescence phenotype, but to a lesser extent than *ima-3* RNAi (Figure S7B), and with an apparent sick phenotype. We next tested for genetic interactions between *akir-1* and *ima-3* by performing *ima-3* RNAi on *akir-1* mutants (Figure 7D). Compared to either *akir-1* mutants or *ima-3* RNAi-treated wild-type animals, the *akir-1* mutant animals exposed to *ima-3* RNAi showed severely delayed growth and reduced body size (Figure 7D), suggesting that AKIR-1 and IMA-3 may cooperate in some cellular processes by acting either in the same complex genetic pathway or in parallel pathways. While it has been suggested that individual importins act as receptors for several different types of protein cargo,^47^ we reasoned that AKIR-1 is likely involved in a more limited role. We therefore used a nuclear localized GFP transgenic reporter strain (*sur-5p::NLS-GFP*)^33^^,^^48^ to investigate whether AKIR-1 affects nuclear import in general. As expected, RNAi against *ima-3* markedly reduced the GFP signal in intestinal nuclei, but *akir-1* RNAi had no effect on the nuclear localization of the GFP reporter suggesting a more specific role for *akir-1* in nuclear import than for importins (Figure 7E). Taken together, our genetic experiments suggest that AKIR-1 acts in the nuclear import of proteasomes cooperating with importins α3 and β1.

## Discussion

Our results reveal a conserved role of the Akirin protein family in regulation of nuclear transport of proteasome and elaborate the knowledge on their complex action in a multicellular organism by presenting a role for *C. elegans* AKIR-1 as a proteasome regulator. Recently, a screen for regulators of the levels of the transcription factor MYC by de Almeida and colleagues identified an unexpected function for human AKIRIN2 as a regulator of the turnover of a subset of nuclear proteins in human cancer cells.^19^ As downregulation of *AKIRIN2* led to reduced nuclear fluorescence signal of tagged proteasome subunits, and as AKIRIN2 was shown with cryoelectron microscopy (cryo-EM) to bind to the 20S proteasome, they suggested that AKIRIN2 acts as a mediator of proteasome nuclear import. We have identified the *AKIRIN2* homolog *akir-1*, in an unbiased genome-wide RNAi screen for novel proteasome regulators in *C. elegans*, and show that AKIR-1 functions in nuclear localization of endogenous proteasomes. More precisely, we demonstrate that animals lacking *akir-1* have reduced levels of nuclear proteasomes, both in intestinal cells and oocytes. Thus, our results reveal the significance of Akirins in regulating nuclear proteasome localization at an organismal level and that this function is conserved between human cells and invertebrates.

The molecular mechanism behind many of the physiological functions of Akirins is believed to be transcriptional regulation due to their interaction with chromatin remodeling complexes and transcription factors,^28^^,^^49^^,^^50^ and as such the effect of AKIR-1 on proteasome localization could be indirect. However, de Almeida and co-workers have shown that the transcript levels of *MYC* or other AKIRIN2 regulated proteins are not altered upon AKIRIN2 knockout.^19^ Similarly, we observed no phenotype resembling the *akir-1* RNAi-induced phenotype upon downregulation of all subunits of the *C. elegans* NuRD and MEC chromatin remodeling complexes, as well as a subunit of RNA polymerase II. A transcription-independent mechanism of AKIR-1 is further supported by our western blot analysis showing no consistent change in proteasome subunit levels or in the amount of assembled proteasome complexes upon *akir-1* depletion, and by a report describing a post-transcriptional effect on the meiosis-linked SYP-1 protein in the *akir-1* mutants.^29^ Thus, our results are in agreement with the presented role of AKIRIN2 and suggest that also AKIR-1 could regulate nuclear transport of proteasomes in *C. elegans*. However, additional mechanisms regulating the subcellular distribution of proteasomes likely exist in *C. elegans*, as we show a reduction, but not a complete loss, of nuclear proteasomes upon both downregulation and loss of *akir-1* in intestinal cells and oocytes. In addition, the *akir-1* null mutants display a developmental defective but viable phenotype, whereas the AKIRIN2 knockout results in apoptosis of human cancer cells.^19^ In human cells, AKIRIN2 is regulating re-importing of proteasomes into the nucleus upon reformation of the nuclear envelope in late mitosis,^19^ but our results reveal that AKIR-1 is also involved in regulating nuclear transport of proteasomes in non-dividing cells, as downregulation of *akir-1* results in reduction of nuclear proteasomes and increase in nuclear accumulation of polyubiquitinated proteins in post-mitotic intestinal cells. Thus, our study suggests a more complex role of AKIR-1 in proteasomal degradation in a multicellular organism.

AKIR-1 contains the highly conserved N-terminal bipartite NLS of the Akirin protein family,^19^^,^^28^ and its nuclear localization has been confirmed in transgenic *C. elegans* expressing AKIR-1::GFP or FLAG-tagged AKIR-1 in several tissues such as intestine, germline, body-wall muscle, and epidermis.^30^^,^^31^^,^^32^ Nuclear transport of proteins is mediated by the importins and exportins of the karyopherin family,^17^ and we show that knockdown of two importin family members, *ima-3* and *imb-1*, mimics the *akir-1* RNAi-induced nuclear accumulation of the polyubiquitin-binding reporter in intestinal cells. The small fluctuation in observed phenotypes between *akir-1* and *importins* knockdowns might derive from variations in protein stability, receptor redundancy, or the existence of more complex proteasome transport mechanisms. We further show that *akir-1* genetically interacts with *ima-3*, either within the same complex pathway or via a parallel pathway. Previously, it has been reported that GFP-tagged AKIR-1 interacts with IMA-3^30^ and that AKIR-1 binds to IMA-2 in a yeast two-hybrid screen.^32^ Interestingly, the *S. cerevisiae* importin α family member SRP1, which is believed to be a homolog of *C. elegans ima-2* and *ima-3*, is required for nuclear import of proteasomes.^18^^,^^46^ Importin 9 (IPO9) and 5 (IPO5) have also been implicated to play a similar role in *D. melanogaster* during spermatogenesis and in human cancer cells.^15^^,^^19^^,^^20^ *C. elegans* has no IPO9 homolog,^46^ and we observed no phenotype with the IPO5 homolog, *imb-3*. Our results suggest that AKIR-1 controls proteasome nuclear import in *C. elegans* together with importin receptors IMA-3 and IMB-1. Interestingly, a study by Bowman and co-workers showed that the function of AKIR-1 on the synaptonemal complex in meiosis is dependent on IMA-2, but not IMA-3.^32^ Together, these studies indicate that AKIR-1 might be involved in regulating nuclear events through interactions with different importins.

In addition to direct interaction between importins and proteasome subunits, it has been shown in yeast that adaptor proteins, such as Sts1 in *S. cerevisiae* and its ortholog Cut8 in *S. pombe*, interact with the 20S/26S proteasome.^21^^,^^22^^,^^23^^,^^24^^,^^25^ A homolog of Sts1/Cut8 has so far been found only in *D. melanogaster*,^24^ but de Almeida and colleagues hypothesized that the human AKIRIN2 could potentially be a functional homolog of Sts1/Cut8, even though lack of sequence homology.^19^ Both AKIRIN2 and Sts1 interact with proteasome, contain protein regions of predicted disorder, and are short-lived proteins.^19^^,^^21^ Our *akir-1* RNAi experiments, starting at L1 or L4 larval stage, consistently displayed a highly penetrant nuclear phenotype in the intestinal polyubiquitin-binding reporter strain, suggesting that AKIR-1 is a short-lived protein also in *C. elegans* intestine. Our AKIR-1 results support that Akirins might function as adaptor proteins in the nuclear transport of proteasomes, but future studies are required to uncover whether AKIR-1 functions in the import of proteasomes to the nucleus or possibly in retaining proteasomes in the nucleus.

Our study further demonstrates a broader role of AKIR-1 in regulation of proteasome function and protein homeostasis in a multicellular organism. Lysates of *akir-1* RNAi-treated animals contained slightly increased *in vitro* proteasome activity, which was mainly due to enhanced activity of the 20S particle. Interestingly, when tissue-level degradation in living animals was monitored, no change was detected in proteasomal degradation of the photoconverted UbG76V-Dendra2 reporter protein at the cellular level in either intestinal cells or body-wall muscle cells. This suggests that the increase in *in vitro* proteasome activity in response to *akir-1* knockdown does not stem from these two cell types, but possibly from another cell type(s) present in the whole animal lysates. Alternatively, as AKIRIN2 has been shown to bind to several gate-forming subunits of the human 20S proteasome and has been suggested to lock the proteasome in an inactive conformation,^19^ we speculate that *akir-1* depletion might directly affect the proteolytic activity of the 20S proteasome or the entry of the short peptide substrate, which does not require unfolding by the 19S, into the 20S proteasome in the *in vitro* assay. Remarkably, when we determined proteasomal degradation rate at the subcellular level, i.e., by separately measuring degradation in the nucleus and cytoplasm of intestinal cells, the *akir-1* RNAi animals showed a clear increase in the nuclear to cytoplasmic fluorescence ratio of photoconverted UbG76V-Dendra2, revealing that downregulation of *akir-1* slows proteasomal degradation in the nucleus. This reduced degradation capacity in the intestinal nucleus reflects the decreased nuclear localization of the proteasome. Together, our *in vivo* data suggest that downregulation of *akir-1* causes a subcellular redistribution of proteasomes and proteasomal degradation capacity in the *C. elegans* intestine.

We have previously reported tissue-specific variations in proteasome activity and regulatory mechanisms in *C. elegans*.^34^^,^^36^^,^^37^ Interestingly, here we show that although oocytes display a more pronounced reduction in the nuclear proteasome levels compared to intestinal cells, acute accumulation of endogenous polyubiquitinated proteins is not induced in the nuclei of these cells upon loss of *akir-1*. Cell type-specific differences in proteasomal substrates could potentially contribute to the different response in the nuclear accumulation of polyubiquitinated proteins between oocytes and intestinal cells. It has also been reported that dissected gonads from young *D. melanogaster* flies display elevated proteasome capacity compared to somatic tissues,^51^^,^^52^ which could contribute to a faster protein turnover in oocytes. Additionally, it has been reported in human cells and *C. elegans* that ubiquitinated proteins are exported from the nucleus to the cytoplasm through a UBIN-POST system (UBIN, a ubiquitin-associated [UBA] domain-containing protein; POST, a polyubiquitinated substrate transporter) as a response to proteasome inhibitor treatment.^12^ As we occasionally observed an accumulation of polyubiquitin-positive staining at the rim of the nuclear membrane in oocytes of *akir-1*-depleted animals, we speculated that the *akir-1* depletion-induced strong reduction in nuclear proteasomes could perhaps mimic a proteasome inhibition at this subcellular compartment and thereby potentially result in nuclear export of polyubiquitinated proteins in oocytes. We tested this hypothesis by separate or combined RNAi knockdown of the *C. elegans* homologs of UBIN and POST, *ubql-1* and *F36D4.5,* respectively, in the *akir-1(gk528)* mutant, but we did not detect nuclear accumulation of polyubiquitinated proteins in oocytes, or any increase in polyubiquitinated proteins in the intestinal cell nuclei (data not shown). Our results imply that the UBIN-POST system would not be involved in the *akir-1*-mediated tissue-specific effect on polyubiquitinated proteins. Due to the complexity of proteins involved in nuclear transport and their crucial roles in various physiological events, dissecting the tissue-specific molecular mechanisms by which AKIR-1 regulates the subcellular localization of polyubiquitinated proteins in *C. elegans* is not trivial. Thus, more in-depth future studies are required.

In addition to oocytes, body-wall muscle cells also responded differently to *akir-1* knockdown compared to the intestinal cells. In the body-wall muscle cells, we observed a later onset of the phenotype, as a slight nuclear accumulation of polyubiquitin-binding reporter was detected only in the next (F_1_) generation after continuous exposure to *akir-1* RNAi treatment. It is unlikely that this later onset is caused by a variation in RNAi efficiency based on the use of an RNAi-sensitive strain and as we have previously shown efficient RNAi capacity in both body-wall muscle cells and intestinal cells.^33^ This could be due to tissue-specific variation in proteasomal degradation rate, as we have demonstrated slower degradation in the body-wall muscle cells compared to intestinal cells.^34^^,^^37^ We speculate that due to the slower proteasomal degradation rate, body-wall muscle cells maintain functional AKIR-1 proteins longer after *akir-1* knockdown compared to intestinal cells. Given the variable effects of AKIR-1 depletion, future studies are required to decipher the contribution of individual molecular mechanisms to the tissue-specific phenotypes.

Overall, our AKIR-1 study demonstrates that the role of Akirins in regulating nuclear proteasome localization is conserved between *C. elegans* and human cells and that Akirin family members can interact with several nuclear transport proteins. Importantly, our results reveal that *akir-1* depletion causes differential outcomes on accumulation of polyubiquitinated proteins in tissues. Lastly, our study suggests a broader role for Akirins in health span regulation and maintenance of cellular protein homeostasis, with a potential tissue-specific impact in multicellular organisms.

### Limitations of study

This study utilizes our new and previously generated fluorescent reporter systems^34^^,^^36^^,^^37^^,^^38^^,^^42^ to uncover cell- and tissue-specific aspects of *akir-1* depletion on the *in vivo* function of the UPS. Unfortunately, the expression of our current fluorescent polyubiquitin-binding reporter and photoconvertible UPS activity reporter is yet restricted to a few cell types, excluding, e.g., germ cells, which limits a more comprehensive comparative *in vivo* analysis of proteasome function.

## STAR★Methods

### Key resources table

**Table:** 

REAGENT or RESOURCE	SOURCE	IDENTIFIER
**Antibodies**		

MCP231	Enzo Life Sciences	Cat# BML-PW8195; RRID: AB_10541045
FK1	StressMarq Biosciences	Cat# SMC-213; RRID: AB_2699340
alpha-tubulin	Sigma-Aldrich	Cat# T5168; RRID: AB_477579
HRP-conjugated anti-mouse IgM	Millipore	Cat# 401225-2ML; RRID: AB_437770
HRP-conjugated anti-mouse IgG	Promega	Cat# W4021; RRID: AB_430834
Alexa Fluor 594 conjugated anti-mouse IgM	Thermo Fisher Scientific	Cat# A-21044; RRID: AB_2535713
Alexa Fluor 594 conjugated anti-mouse IgG	Thermo Fisher Scientific	Cat# A-11005; RRID: AB_2534073 or Cat# R37121; RRID: AB_2556549

**Bacterial and virus strains**		

OP50 *Escherichia coli*	CGC	WB Cat# WBStrain00041969
Biological samples	N/A	N/A

**Chemicals, peptides, and recombinant proteins**		

ampicillin	Merck KGaA	Cat# A0166
tetracycline	Merck KGaA	Cat# T7660
isopropyl β-D-1-thiogalactopyranoside	Merck KGaA	Cat# I6758
N-ethylmaleimide	Merck KGaA	Cat# E3876
MG-132	Peptides International	Cat# IZL-3175-v
Pierce Protease Inhibitor Mini Tablet	Thermo Fisher Scientific	Cat# A32955
fluorogenic proteasome substrate succinyl-leu-leu-val-tyr-7-amino-4-methylcoumarin	Bachem	Cat# l.1395
ATP	Sigma-Aldrich	Cat# A3377
Ponceau S staining	Sigma-Aldrich	Cat# P3504
levamisole hydrocloride	Merck KGaA	Cat# T7660
SlowFade Diamond Antifade Mountant	Thermo Fisher Scientific	Cat# S36967
Hoechst 33342	Merck KGaA	Cat# B2261

**Critical commercial assays**		

Nucleospin RNA kit	MACHEREY-NAGEL GmbH & Co. KG	Cat# 740955
Maxima First Strand cDNA synthesis kit for RT-qPCR	Thermo Fisher Scientific	Cat# K1641
Maxima SYBR Green/Rox qPCR Master Mix	Thermo Fisher Scientific	Cat# K0221
Colloidal Blue staining kit	Thermo Fisher Scientific	Cat# LC6025

**Experimental models: Organisms/strains**		

C. elegans: Strain N2 Bristol	CGC	WBStrain00000001
C. elegans: Strain VC1056[*akir-1(gk528)I*]	CGC	WBStrain00036286
C. elegans: Strain NL2099[*rrf-3(pk1426)II*]	CGC	WBStrain00028995
C. elegans: Strain YD3[*xzEx3[unc-54p::UbG76V::Dendra2]*]	(Hamer et al.)^37^	WBStrain00046811
C. elegans: Strain YD27[*xzEx27[vha-6p::UbG76V::Dendra2]*]	(Li et al.)^38^	WBStrain00049245
C. elegans: Strain YD90[*xzIs1[vha-6p::UIM2::ZsProSensor]*]	(Matilainen et al.)^34^	WBStrain00046813
C. elegans: Strain GR1702[*Is1[sur-5p::NLS-GFP]*]	(Mikkonen et al.)^33^	N/A
C. elegans: Strain CER395*[ubh-4(cer68[ubh-4::egfp])II]*	Julián Cerón (Martinez-Fernandez et al.; Vicencio et al.)^42^^,^^43^	N/A
C. elegans: Strain YD116[*rrf-3(pk1426);xzIs2[unc-54p::UIM2::ZsProSensor]*]	(Jha & Holmberg)^35^	N/A
C. elegans: Strain YD114[*xzIs2[unc-54p::UIM2::ZsProSensor]*]	(Pispa et al.)^36^	WBStrain00049246
C. elegans: Strain YD25*[xzEx25[vha-6p::Dendra2]]*	(Li et al.)^38^	WBStrain00049244
C. elegans: Strain YD117[*xzEx113[rpt-5p::GFP::RPT-5]*]	This paper	N/A

**Oligonucleotides**		

*akir-1*: 5′-gatatgcgaacgtctgctca; 5′-ggaatagtcatccccagtgc	This paper	N/A
*act-1:* 5′-tcggtatgggacagaaggac; 5′-catcccagttggtgacgata	This paper	N/A
*cdc-42:* 5′-ctgctggacaggaagattacg; 5′-ctcggacattctcgaatgaag	This paper	N/A
*pmp-3:* 5′-gttcccgtgttcatcactcat, 5′-acaccgtcgagaagctgtaga	This paper	N/A

**Recombinant DNA**		

Plasmid: pPD118.25	Addgene	Addgene plasmid #1593; http://n2t.net/addgene:1593; RRID:Addgene_1593

**Software and algorithms**		

Fiji software	(Schindelin et al.)^58^	
Microscopy Image Browser software	(Belevich et al.)^60^	
Zeiss Zen 2 software	Zeiss	
Adobe Photoshop	Adobe	
Microsoft Excel 2016 spreadsheet	Microsoft	
RStudio software	RStudio	

**Other**		

J. Ahringer RNAi library	Source BioScience	
sequencing	Eurofins Genomics	
OASIS 2 online tool	(Han et al.)^59^	

### Resource availability

#### Lead contact

Further information and requests for resources and reagents should be directed to and will be fulfilled by the lead contact, Carina I. Holmberg (carina.holmberg@helsinki.fi).

#### Materials availability

The transgenic *C. elegans* strain generated in this study will be available upon request.

#### Data and code availability

• All data reported in this paper will be shared by the lead contact upon request.
• This paper does not report original code.
• Any additional information required to reanalyze the data reported in this paper is available from the lead contact upon request.

### Experimental models and study participants

#### C. elegans strains

All strains were cultured as previously described^53^ at 20°C on nematode growth medium (NGM) plates seeded with OP50 *Escherichia coli* bacteria. N2 Bristol, VC1056[*akir-1(gk528)I*], and NL2099[*rrf-3(pk1426)II*] strains were obtained from the Caenorhabditis Genetic Center (CGC, Minneapolis, MN, USA). The *akir-1* mutant allele *gk528* was backcrossed three times with wild-type N2 animals. Following strains were also used: YD3[*xzEx3[unc-54p::UbG76V::Dendra2]*],^37^ YD27[*xzEx27[vha-6p::UbG76V::Dendra2]*],^38^ YD90[*xzIs1[vha-6p::UIM2::ZsProSensor]*],^34^ GR1702[*Is1[sur-5p::NLS-GFP]*],^33^ CER395*[ubh-4(cer68[ubh-4::egfp])II]*,^42^^,^^43^ YD116[*rrf-3(pk1426);xzIs2[unc-54p::UIM2::ZsProSensor]*],^35^ and YD114[*xzIs2[unc-54p::UIM2::ZsProSensor]*].^36^ To generate YD117[*xzEx113[rpt-5p::GFP::RPT-5]*] reporter strain, GFP::RPT-5 translational fusion construct was created by substituting the let-858 promoter of the GFP vector pPD118.25 (L3786 was a gift from Andrew Fire; Addgene plasmid #1593; http://n2t.net/addgene:1593; RRID:Addgene_1593; Watertown, MA, USA) with a 2010-bp putative rpt-5 promoter sequence, mutating the GFP stop codon, and replacing the let858 3′ UTR with a 1747-bp sequence covering the 1504-bp rpt-5 coding region with introns and the following 183-bp 3′ UTR region. To create extrachromosomal transgenic line, the plasmid DNA (120–180 ng/μL) was injected into gonads of young N2 adults along with *Pmyo-2*::CFP (20 ng/μL) marker. Unless otherwise stated, animals were age-synchronized with bleach solution (0.25 M NaOH and 0.54% NaOCl), and L1 larvae (day 1) were placed on bacteria-seeded plates prior being collected at first day of adulthood (day 4). Due to slow growth age-synchronized *akir-1* mutant L1 larvae were placed on bacteria-seeded plates 10–17 h before the age-synchronized N2 L1 larvae. Both N2 and *akir-1* mutant animals were collected at first day of adulthood (day 4 with N2 and day 5 with *akir-1* mutant animals). The experiments were done either with a hermaphrodite population, with males existing at a frequency of <0.2% in N2^54^ and 0.9% in *akir-1(gk528)*,^29^ or with singly picked hermaphrodites.

### Method details

#### C. elegans RNA interference (RNAi)

Select gene RNAi was performed using the feeding method as described earlier^55^ with a few changes. The HT115(DE3) bacterial strain carrying the empty *pL4440* cloning vector was used as a control. A single bacteria colony was cultured overnight at 37°C in Luria broth (LB) medium containing 100 μg/mL ampicillin (Merck KGaA Cat# A0166, Darmstadt, Germany) and 12.5 μg/mL tetracycline (Merck KGaA Cat# T7660). The culture was diluted 100-fold and allowed to grow in 2xYT medium containing ampicillin and tetracycline in above-mentioned concentrations until the absorbance at 600 nm (OD600) reached 0.4. The double-stranded RNA expression was induced using 0.4 mM isopropyl β-D-1-thiogalactopyranoside, IPTG (Merck KGaA Cat# I6758) and the culture was further grown for 3 h at 37°C. Before adding bacterial cells on RNAi feeding agar plates, cultures were supplemented with additional ampicillin and tetracycline, and the IPTG concentration was increased to 0.8 mM. RNAi feeding agar plates were composed of standard NGM complemented with 100 μg/mL ampicillin, 12.5 μg/mL tetracycline, and 0.4 mM IPTG. For experiments related to quantification of changes in fluorescence of YD90, YD114 or YD116 animals, the RNAi clones were cultured for 6 h at 37°C in 2xYT medium complemented with the above-mentioned antibiotics, induced with 0.8 mM IPTG, and cultured overnight at 37°C prior to plating on the RNAi feeding agar plates. Unless otherwise stated, animals were age-synchronized with bleach treatment, and L1 larvae (day 1) were placed on control or RNAi-seeded feeding plates prior being collected at first day of adulthood (day 4). When *akir-1* mutant animals were exposed to RNAi treatment, age-synchronized L1 larvae were placed on the RNAi plates 10–17 h before the age-synchronized N2 L1 larvae. Both N2 and *akir-1* mutant animals were collected at first day of adulthood (day 4 with N2 and day 5 with *akir-1* mutant animals). RNAi clones for the following genes were used: *akir-1(E01A2.6), hda-1(C53A5.3), let-418(F26F12.7), lin-53(K07A1.12), lin-40(T27C4.4), chd-3(T14G8.1), mep-1(M04B2.1), dcp-66(C26C6.5), ama-1(F36A4.7), ima-1(T19B10.7), ima-2(F26B1.3), ima-3(F32E10.4), imb-2(R06A4.4), imb-3(C53D5.a), T16G12.6, tsr-1(F53G2.6), xpo-1(ZK742.1), xpo-2(Y48G1A.5), xpo-3(C49H3.10); ubql-1(F15C11.2); F36D4.5* (J. Ahringer RNAi library, Source BioScience, Nottingham, UK), or *imb-1(F28B3.8), C35A5.8, Y69A2AR.16* (clones were kind gifts from Dr. Susana Garcia; Vidal ORFeome). All phenotype-inducing RNAi clones as well as most of the no phenotype–inducing clones were confirmed by sequencing (Eurofins Genomics, Ebersberg, Germany).

The experimental setup of our original genome-wide RNAi screen for UPS regulators was slightly different than for the select gene RNAi. Briefly, we used the J. Ahringer RNAi library constructed by Kamath and Ahringer^56^ and commercially obtained from Source BioScience. Bacterial clones were cultured overnight at 37°C in 2xYT medium containing antibiotics without a subsequent culture with IPTG before seeding. Age-synchronized YD90[*xzIs1[vha-6p::UIM2::ZsProSensor]*]^34^ L1 larvae were placed on RNAi seeding plates, and manually scored after three days as young adults for visible changes in fluorescence with Leica MZ16 FA fluorescence stereomicroscope with a GFP Plus filter (Leica Microsystems, Wetzlar, Germany). Animals with delayed or arrested development or otherwise atypical appearance were censored from further study. Further, RNAi clones affecting general protein synthesis, monitored by changes in the fluorescence of YD25*[xzEx25[vha-6p::Dendra2]]* strain,^38^ were excluded as hits.

#### Quantitative real-time PCR (qPCR)

Age-synchronized animals treated with RNAi were harvested in M9 buffer (22 mM KH_2_PO_4_, 41 mM Na_2_HPO_4_, 8.5 mM NaCl, and 19 mM NH_4_Cl) at first day of adulthood and stored at −80°C. RNA was extracted using Nucleospin RNA kit (MACHEREY-NAGEL GmbH & Co. KG Cat# 740955, Düren, Germany) and cDNA synthesis was done with Maxima First Strand cDNA synthesis kit for RT-qPCR (Thermo Fisher Scientific Cat# K1641, Waltham, MA, USA). Quantitative real-time PCR was performed with Maxima SYBR Green/Rox qPCR Master Mix (Thermo Fisher Scientific Cat# K0221) and LightCycler 480 quantitative PCR machine (Roche Diagnostics International AG, Rotkreuz, Switzerland). The qPCR data were normalized to the expression of three reference genes (*act-1*, *cdc-42*, and *pmp-3*), and comparative Ct (ΔΔCt) method was used to quantify relative expressions of *akir-1* mRNA. Oligo sequences were as follows: 5′-gatatgcgaacgtctgctca, 5′-ggaatagtcatccccagtgc (*akir-1*); 5′-tcggtatgggacagaaggac, 5′-catcccagttggtgacgata (*act-1*); 5′-ctgctggacaggaagattacg, 5′-ctcggacattctcgaatgaag (*cdc-42*); and 5′-gttcccgtgttcatcactcat, 5′-acaccgtcgagaagctgtaga (*pmp-3*).

#### Western blot analysis

Age-synchronized animals fed with OP50 or RNAi bacteria were harvested in M9 buffer at first day of adulthood and animal pellets were stored at −80°C. Pellets were lysed using lysis buffer (50 mM HEPES, 150 mM NaCl, 5 mM EDTA) supplemented with 20 mM N-ethylmaleimide, NEM (Merck KGaA Cat# E3876) and 10 μM MG-132 (Peptides International Cat# IZL-3175-v, Louisville, KY, USA) to inhibit deubiquitination and degradation of ubiquitin-conjugated proteins, respectively. In addition, lysis buffer was supplemented with a Pierce Protease Inhibitor Mini Tablet (Thermo Fisher Scientific Cat# A32955). Lysed samples were separated on an SDS (sodium dodecyl sulfate) polyacrylamide gel and blotted onto a nitrocellulose membrane using Trans-Blot Turbo Transfer System (Bio-Rad Laboratories, Hercules, California, USA). Antibodies against proteasome 20S α-subunits, MCP231 (Enzo Life Sciences Cat# BML-PW8195, RRID:AB_10541045, New York, NY, USA) in 1:1000 dilution, polyubiquitinated proteins, FK1 (StressMarq Biosciences Cat# SMC-213, RRID:AB_2699340, Victoria, Canada) in 1:500 dilution, and alpha-tubulin (Sigma-Aldrich Cat# T5168, RRID:AB_477579) in 1:10000 dilution were used in blotting. The following HRP-conjugated anti-mouse IgM (Millipore Cat# 401225-2ML, RRID:AB_437770) and IgG (Promega Cat# W4021, RRID:AB_430834, Madison, WI, USA) antibodies in 1:10 000 dilutions were used. ECL signals were visualized with Odyssey FC Imaging System (LI-COR, Lincoln, NE, USA), and quantified with Image Studio software (Li-COR). Alpha-tubulin signal was used for normalization.

#### Native gel analysis

Age-synchronized animals fed with RNAi bacteria were harvested in M9 buffer at first day of adulthood and animal pellets were stored at −80°C. Animal pellets were lysed using a Dounce homogenizer and native gel lysis buffer (50 mM Tris-HCL (pH 8.0), 5 mM Mg_2_Cl_2_, 0.5 mM EDTA, and 1 mM ATP (Sigma-Aldrich Cat# A3377)). Native gel electrophoresis and the in-gel proteasome activity assay were performed as earlier reported with a few exceptions.^34^^,^^57^ Gels were run in an ice bath for 30 min at 20 mA and then for 2 h at 40 mA. The gels were either blotted onto a nitrocellulose membrane prior protein detection with the antibody against proteasome 20S α-subunits or incubated in developing buffer containing 160 μM of fluorogenic proteasome substrate succinyl-leu-leu-val-tyr-7-amino-4-methylcoumarin, suc-LLVY AMC (Bachem Cat# l.1395, Bubendorf, Switzerland) and imaged either with MultiImage Light Cabinet using FluorChem 8900 software (Alpha Innotech Corporation, San Leandro, CA, USA) or Gel Doc XR + System with Image Lab Software (Bio-Rad). Total protein normalization was performed from native gels using Colloidal Blue staining kit (Thermo Fisher Scientific Cat# LC6025) after in-gel proteasome activity assay and from nitrocellulose membranes using Ponceau S staining (Sigma-Aldrich Cat# P3504) after blot transfer. Image analysis were made with Fiji software.^58^

#### Immunofluorescence with dissected C. elegans

Age-synchronized animals cultured either on OP50 seeded plates or RNAi feeding plates were harvested at first day of adulthood in M9 buffer. Animals were transferred onto a glass dish and dissected using 27-gauge syringe needles. To immobilize the animals 1 mM levamisole hydrocloride (Merck KGaA Cat# T7660) was used prior making incision close to the pharynx forcing the intestine and gonad to extrude. Dissected animals were fixed with 2xRFB (160 mM KCL, 40 mM NaCl, 20 mM EGTA, 10 mM spermidine, 30 mM PIPES pH 7.4, 50% methanol, and 1% formaldehyde). Additional 100% methanol fixation for 1 min was utilized when antibody against the proteasome 20S alpha subunits was used. Fixed dissected animals were permeabilized using 0.5 or 1% Triton X-100 in PBS and mounted with SlowFade Diamond Antifade Mountant (Thermo Fisher Scientific Cat# S36967). Antibodies against polyubiquitinated proteins, FK1 (RRID:AB_2699340) and the proteasome 20S alpha subunits, MCP231 (RRID:AB_10541045) were used in 1:200 dilution. The following Alexa Fluor 594 conjugated anti-mouse IgM (Thermo Fisher Scientific Cat# A-21044, RRID:AB_2535713) and IgG (Thermo Fisher Scientific Cat# A-11005, RRID:AB_2534073 or Cat# R37121, RRID:AB_2556549) secondary antibodies in 1:200 and 1:100 dilution respectively were used for visualization. The nuclear immunostaining was visually estimated as none, when nuclear staining intensity was similar or less compared to staining intensity in the cytoplasm. A higher nuclear staining intensity compared to the cytoplasmic staining was further estimated visually as weak or strong. DNA was stained with 4 μg/ml Hoechst 33342 (Merck KGaA Cat# B2261).

#### Lifespan assays and progeny counts

Lifespan experiments were performed at 20°C. Age-synchronized animals were plated on RNAi feeding plates as L1 larvae (day 1). Animals were transferred to a new plate, first every second day, and then every few days after they stopped producing offspring. Animals were checked daily, and animals failing to respond to a gentle prod with a platinum worm pick were classified as dead. Animals crawling off the plate, dying of an extruded gonad, or carrying internally hatched offspring were censored at the time of their death. Basic survival analysis was performed using an online tool, OASIS 2.^59^ Data from all three separate lifespan experiments were combined and used for analysis as one dataset. For counting progeny, age-synchronized animals were plated individually on RNAi feeding plates as L1 larvae. Animals were moved to fresh RNAi feeding plates every day, until they stopped producing offspring. Total viable offspring per animal was counted, censoring the offspring of animals that crawled off the plate, died of internally hatched offspring or an extruded gonad before finishing egg-laying.

#### Microscopy

Age-synchronized animals were imaged at first day of adulthood unless otherwise mentioned. Animals were mounted on 3% agarose pads and immobilized with 1 mM levamisole. Group of 5–15 live animals were imaged with a Zeiss Axio Imager 2 upright wide-field light microscope and a Zeiss EC Plan Neofluar numerical aperture (NA) 10 x 0.3 objective or a Plan Apochromat NA 20× 0.8 objective, or with an LSM 780 inverted confocal microscope and a Plan-Neofluor NA 40 x 1.3 or a Plan-Apochromat NA 63 x 1.40 objective, or with a Zeiss LSM 880 inverted confocal microscope and a Plan-Apochromat NA 40 x 1.40 or NA 63 x 1.4 objective (Zeiss, Oberkochen, Germany). All the microscopes run a Zeiss Zen 2 software (Zeiss). For Hoechst nuclear staining, age-synchronized animals were washed with M9, fixed for 2 min using 100% methanol, and permeabilized either with 0.5% or 1% Triton X-100 in PBS. DNA was labeled with 4 μg/ml Hoechst 33342 stain (Merck KGaA Cat# B2261). For photoconversion of the tissue-specific UbG76V-Dendra2, green Dendra2 protein was converted to red using 405-nm UV light. Degradation of the red signal was followed 16–18 (intestine) or 24 (muscle) hours later. For determining the nuclear/cytoplasmic ratio of the red UbG76V-Dendra2 signal, images were taken with the Zeiss LSM 880 inverted confocal microscope and the Plan-Apochromat NA 63 x 1.4 objective, and mean fluorescent intensity of both the nuclei and cytoplasm were quantified using Microscopy Image Browser software.^60^

#### Fluorescence signal quantification

Fluorescence signal was quantified from the original, unmodified TIFF file format images with Fiji software or from the original CZI file format with Zeiss Zen 2 software (Zeiss). In Fiji, sliding paraboloid algorithm was used to subtract background. The mean fluorescence intensity was quantified using an adequate threshold to select fluorescence signal and using the measure function. The brightness of the fluorescence signal has been adjusted in some images using Fiji or Adobe Photoshop (Adobe, San Jose, CA, USA), and all images presented together for comparison were adjusted similarly, unless otherwise stated. In Zeiss Zen 2, profile tab was used to measure fluorescence intensity profiles along a line intersecting the cytoplasm and the nucleus. Hoechst signal was used to determine the nuclear fluorescence. To be able to compare nuclear fluorescence intensity profiles in different cells and between different treatments the nuclear signal along the profiling line was set to start after the same distance. To normalize the fluorescence intensity profiles in control treatment the signal intensity at the starting point was set to 1. The mean intensity signal of the control treatment at the starting point was used to determine the relative fold change in the fluorescence profiles upon the treatment.

### Quantification and statistical analysis

Quantifications are described in the previous sections under the method details section. The statistical significance was determined using Welch’s t-test (two-tailed distribution and unequal variance) using Microsoft Excel 2016 spreadsheet (Microsoft, Redmond, WA, USA). For lifespan analysis, statistical significance was determined with a Mantel-Cox (log rank) test using RStudio software (RStudio, Boston, MA, USA) and the lifespan data used was stratified into three independent experiments.

## References

[1] Kleiger G., Mayor T. (2014). Perilous journey: a tour of the ubiquitin-proteasome system. Trends Cell Biol..

[2] Sakata E., Eisele M.R., Baumeister W. (2021). Molecular and cellular dynamics of the 26S proteasome. Biochim. Biophys. Acta, Proteins Proteomics.

[3] Demasi M., da Cunha F.M. (2018). The physiological role of the free 20S proteasome in protein degradation: A critical review. Biochim. Biophys. Acta Gen. Subj..

[4] Budenholzer L., Cheng C.L., Li Y., Hochstrasser M. (2017). Proteasome Structure and Assembly. J. Mol. Biol..

[5] Wójcik C., DeMartino G.N. (2003). Intracellular localization of proteasomes. Int. J. Biochem. Cell Biol..

[6] Enenkel C., Kang R.W., Wilfling F., Ernst O.P. (2022). Intracellular localization of the proteasome in response to stress conditions. J. Biol. Chem..

[7] von Mikecz A. (2006). The nuclear ubiquitin-proteasome system. J. Cell Sci..

[8] Geng F., Wenzel S., Tansey W.P. (2012). Ubiquitin and proteasomes in transcription. Annu. Rev. Biochem..

[9] Bach S.V., Hegde A.N. (2016). The proteasome and epigenetics: zooming in on histone modifications. Biomol. Concepts.

[10] Enenkel C., Lehmann A., Kloetzel P.M. (1998). Subcellular distribution of proteasomes implicates a major location of protein degradation in the nuclear envelope-ER network in yeast. EMBO J..

[11] Dang F.W., Chen L., Madura K. (2016). Catalytically Active Proteasomes Function Predominantly in the Cytosol. J. Biol. Chem..

[12] Hirayama S., Sugihara M., Morito D., Iemura S.I., Natsume T., Murata S., Nagata K. (2018). Nuclear export of ubiquitinated proteins via the UBIN-POST system. Proc. Natl. Acad. Sci. USA.

[13] Savulescu A.F., Shorer H., Kleifeld O., Cohen I., Gruber R., Glickman M.H., Harel A. (2011). Nuclear import of an intact preassembled proteasome particle. Mol. Biol. Cell.

[14] Pack C.G., Yukii H., Toh-e A., Kudo T., Tsuchiya H., Kaiho A., Sakata E., Murata S., Yokosawa H., Sako Y. (2014). Quantitative live-cell imaging reveals spatio-temporal dynamics and cytoplasmic assembly of the 26S proteasome. Nat. Commun..

[15] Spits M., Janssen L.J., Voortman L.M., Kooij R., Neefjes A.C.M., Ovaa H., Neefjes J. (2019). Homeostasis of soluble proteins and the proteasome post nuclear envelope reformation in mitosis. J. Cell Sci..

[16] van der Zanden S.Y., Jongsma M.L.M., Neefjes A.C.M., Berlin I., Neefjes J. (2023). Maintaining soluble protein homeostasis between nuclear and cytoplasmic compartments across mitosis. Trends Cell Biol..

[17] Wing C.E., Fung H.Y.J., Chook Y.M. (2022). Karyopherin-mediated nucleocytoplasmic transport. Nat. Rev. Mol. Cell Biol..

[18] Lehmann A., Janek K., Braun B., Kloetzel P.M., Enenkel C. (2002). 20 S proteasomes are imported as precursor complexes into the nucleus of yeast. J. Mol. Biol..

[19] de Almeida M., Hinterndorfer M., Brunner H., Grishkovskaya I., Singh K., Schleiffer A., Jude J., Deswal S., Kalis R., Vunjak M. (2021). AKIRIN2 controls the nuclear import of proteasomes in vertebrates. Nature.

[20] Palacios V., Kimble G.C., Tootle T.L., Buszczak M. (2021). Importin-9 regulates chromosome segregation and packaging in Drosophila germ cells. J. Cell Sci..

[21] Budenholzer L., Breckel C., Hickey C.M., Hochstrasser M. (2020). The Sts1 nuclear import adapter uses a non-canonical bipartite nuclear localization signal and is directly degraded by the proteasome. J. Cell Sci..

[22] Chen L., Romero L., Chuang S.M., Tournier V., Joshi K.K., Lee J.A., Kovvali G., Madura K. (2011). Sts1 plays a key role in targeting proteasomes to the nucleus. J. Biol. Chem..

[23] Takeda K., Tonthat N.K., Glover T., Xu W., Koonin E.V., Yanagida M., Schumacher M.A. (2011). Implications for proteasome nuclear localization revealed by the structure of the nuclear proteasome tether protein Cut8. Proc. Natl. Acad. Sci. USA.

[24] Takeda K., Yanagida M. (2005). Regulation of nuclear proteasome by Rhp6/Ubc2 through ubiquitination and destruction of the sensor and anchor Cut8. Cell.

[25] Tatebe H., Yanagida M. (2000). Cut8, essential for anaphase, controls localization of 26S proteasome, facilitating destruction of cyclin and Cut2. Curr. Biol..

[26] Weberruss M.H., Savulescu A.F., Jando J., Bissinger T., Harel A., Glickman M.H., Enenkel C. (2013). Blm10 facilitates nuclear import of proteasome core particles. EMBO J..

[27] Artigas-Jerónimo S., Villar M., Cabezas-Cruz A., Valdés J.J., Estrada-Peña A., Alberdi P., de la Fuente J. (2018). Functional Evolution of Subolesin/Akirin. Front. Physiol..

[28] Bosch P.J., Peek S.L., Smolikove S., Weiner J.A. (2020). Akirin proteins in development and disease: critical roles and mechanisms of action. Cell. Mol. Life Sci..

[29] Clemons A.M., Brockway H.M., Yin Y., Kasinathan B., Butterfield Y.S., Jones S.J.M., Colaiácovo M.P., Smolikove S. (2013). akirin is required for diakinesis bivalent structure and synaptonemal complex disassembly at meiotic prophase I. Mol. Biol. Cell.

[30] Polanowska J., Chen J.X., Soulé J., Omi S., Belougne J., Taffoni C., Pujol N., Selbach M., Zugasti O., Ewbank J.J. (2018). Evolutionary plasticity in the innate immune function of Akirin. PLoS Genet..

[31] Bowman R., Balukoff N., Clemons A., Koury E., Ford T., Baxi K., Egydio de Carvalho C., Smolikove S. (2020). Akirin Is Required for Muscle Function and Acts Through the TGF-beta Sma/Mab Signaling Pathway in Caenorhabditis elegans Development. G3 (Bethesda).

[32] Bowman R., Balukof N., Ford T., Smolikove S. (2019). A Novel Role for alpha-Importins and Akirin in Establishment of Meiotic Sister Chromatid Cohesion in Caenorhabditis elegans. Genetics.

[33] Mikkonen E., Haglund C., Holmberg C.I. (2017). Immunohistochemical analysis reveals variations in proteasome tissue expression in C. elegans. PLoS One.

[34] Matilainen O., Arpalahti L., Rantanen V., Hautaniemi S., Holmberg C.I. (2013). Insulin/IGF-1 signaling regulates proteasome activity through the deubiquitinating enzyme UBH-4. Cell Rep..

[35] Jha S., Holmberg C.I. (2020). Tissue-Specific Impact of Autophagy Genes on the Ubiquitin-Proteasome System in C. elegans. Cells.

[36] Pispa J., Matilainen O., Holmberg C.I. (2020). Tissue-specific effects of temperature on proteasome function. Cell Stress Chaperones.

[37] Hamer G., Matilainen O., Holmberg C.I. (2010). A photoconvertible reporter of the ubiquitin-proteasome system in vivo. Nat. Methods.

[38] Li X., Matilainen O., Jin C., Glover-Cutter K.M., Holmberg C.I., Blackwell T.K. (2011). Specific SKN-1/Nrf stress responses to perturbations in translation elongation and proteasome activity. PLoS Genet..

[39] Hamazaki J., Iemura S.I., Natsume T., Yashiroda H., Tanaka K., Murata S. (2006). A novel proteasome interacting protein recruits the deubiquitinating enzyme UCH37 to 26S proteasomes. EMBO J..

[40] Qiu X.B., Ouyang S.Y., Li C.J., Miao S., Wang L., Goldberg A.L. (2006). hRpn13/ADRM1/GP110 is a novel proteasome subunit that binds the deubiquitinating enzyme, UCH37. EMBO J..

[41] Yao T., Song L., Xu W., DeMartino G.N., Florens L., Swanson S.K., Washburn M.P., Conaway R.C., Conaway J.W., Cohen R.E. (2006). Proteasome recruitment and activation of the Uch37 deubiquitinating enzyme by Adrm1. Nat. Cell Biol..

[42] Martinez-Fernandez C., Jha S., Aliagas E., Holmberg C.I., Nadal E., Ceron J. (2023). BAP1 Malignant Pleural Mesothelioma Mutations in Caenorhabditis elegans Reveal Synthetic Lethality between ubh-4/BAP1 and the Proteasome Subunit rpn-9/PSMD13. Cells.

[43] Vicencio J., Martínez-Fernández C., Serrat X., Cerón J. (2019). Efficient Generation of Endogenous Fluorescent Reporters by Nested CRISPR in Caenorhabditis elegans. Genetics.

[44] Altun Z.F., Hall D.H. (2009). Alimentary system, intestine. https://www.wormatlas.org/hermaphrodite/intestine/Intframeset.html.

[45] Passannante M., Marti C.O., Pfefferli C., Moroni P.S., Kaeser-Pebernard S., Puoti A., Hunziker P., Wicky C., Müller F. (2010). Different Mi-2 Complexes for Various Developmental Functions in Caenorhabditis elegans. PLoS One.

[46] Adam S.A. (2009). The nuclear transport machinery in Caenorhabditis elegans: A central role in morphogenesis. Semin. Cell Dev. Biol..

[47] Kimura M., Morinaka Y., Imai K., Kose S., Horton P., Imamoto N. (2017). Extensive cargo identification reveals distinct biological roles of the 12 importin pathways. Elife.

[48] Yochem J., Gu T., Han M. (1998). A new marker for mosaic analysis in Caenorhabditis elegans indicates a fusion between hyp6 and hyp7, two major components of the hypodermis. Genetics.

[49] Goto A., Matsushita K., Gesellchen V., El Chamy L., Kuttenkeuler D., Takeuchi O., Hoffmann J.A., Akira S., Boutros M., Reichhart J.M. (2008). Akirins are highly conserved nuclear proteins required for NF-kappaB-dependent gene expression in drosophila and mice. Nat. Immunol..

[50] Nowak S.J., Aihara H., Gonzalez K., Nibu Y., Baylies M.K. (2012). Akirin links twist-regulated transcription with the Brahma chromatin remodeling complex during embryogenesis. PLoS Genet..

[51] Fredriksson Å., Johansson Krogh E., Hernebring M., Pettersson E., Javadi A., Almstedt A., Nyström T. (2012). Effects of aging and reproduction on protein quality control in soma and gametes of Drosophila melanogaster. Aging Cell.

[52] Tsakiri E.N., Sykiotis G.P., Papassideri I.S., Gorgoulis V.G., Bohmann D., Trougakos I.P. (2013). Differential regulation of proteasome functionality in reproductive vs. somatic tissues of Drosophila during aging or oxidative stress. FASEB J..

[53] Brenner S. (1974). The genetics of Caenorhabditis elegans. Genetics.

[54] Hodgkin J., Doniach T. (1997). Natural variation and copulatory plug formation in Caenorhabditis elegans. Genetics.

[55] Timmons L., Court D.L., Fire A. (2001). Ingestion of bacterially expressed dsRNAs can produce specific and potent genetic interference in Caenorhabditis elegans. Gene.

[56] Kamath R.S., Ahringer J. (2003). Genome-wide RNAi screening in Caenorhabditis elegans. Methods.

[57] Elsasser S., Schmidt M., Finley D. (2005). Characterization of the proteasome using native gel electrophoresis. Method Enzymol.

[58] Schindelin J., Arganda-Carreras I., Frise E., Kaynig V., Longair M., Pietzsch T., Preibisch S., Rueden C., Saalfeld S., Schmid B. (2012). Fiji: an open-source platform for biological-image analysis. Nat. Methods.

[59] Han S.K., Lee D., Lee H., Kim D., Son H.G., Yang J.S., Lee S.J.V., Kim S. (2016). OASIS 2: online application for survival analysis 2 with features for the analysis of maximal lifespan and healthspan in aging research. Oncotarget.

[60] Belevich I., Joensuu M., Kumar D., Vihinen H., Jokitalo E. (2016). Microscopy Image Browser: A Platform for Segmentation and Analysis of Multidimensional Datasets. PLoS Biol..

